# Network Pharmacology-Based Analysis of the Underlying Mechanism of Huajiao for Pain Relief

**DOI:** 10.1155/2021/5526132

**Published:** 2021-04-04

**Authors:** Mingquan Wu, Hong-Ling Du, Xu Zhou, Wei Peng, Limei Liu, Zhong Zhang, He Tu

**Affiliations:** ^1^Department of Pharmacy, Sichuan Orthopedic Hospital, Chengdu, Sichuan, China; ^2^College of Pharmacy, Chengdu University of Traditional Chinese Medicine, Chengdu, Sichuan, China

## Abstract

**Objective:**

Pain is a common symptom among patients, and pain management is an important clinical practice topic. The mechanism of Huajiao (HJ; *Zanthoxylum bungeanum* Maxim.) and its effective components for treating pain was explored using network pharmacology and molecular docking to verify its pain relief function in traditional medical practice.

**Methods:**

HJ's components were collected via the Traditional Chinese Medicine Systems Pharmacology platform and published studies. HJ-associated target proteins were predicted using the drug similarity rule via Swiss Target Prediction. Online Mendelian Inheritance in Man was used to search for pain-related genes and proteins, and the Database of Interacting Proteins was used to obtain the human interactive target proteins. The compound-target-disease network of HJ for pain relief was constructed with protein-protein interaction networks. The obtained target proteins were uploaded on the Database for Annotation, Visualization, and Integrated Discovery to annotate, visualize, and integrally discover the related signaling pathway, and semiflexible molecular docking by Autodock Vina was applied to verify the potential mechanism.

**Results:**

A total of 157 molecules in HJ were obtained, and the top 20 active components or active groups were mainly focused on the amide alkaloids (e.g., [6RS]-[2E,7E,9E]-6-hydroxy-N-[2-hydroxy-2-methylpropyl]-11-oxo-2,7,9-dodecatrienamide and [2E,7E,9E]-N-[2-hydroxy-2-methylpropyl]-11-ethoxy-6-hydroxy-dodeca-2,7,9-trienamide). Also, the 66 main targets were filtered from 746 predicted targets and 928 pain-related targets through module Network Analyzer in Cytoscape 3.6.0. Finally, there were 3 critical signaling pathways, including mitogen-activated protein kinase, phosphoinositide 3-kinase-protein kinase B-mammalian target of rapamycin, and I*κ*B kinase-nuclear factor *κ*B-cyclooxygenase 2 based on integrated discovery with 54 enriched signaling pathways.

**Conclusions:**

HJ is used as a pain relief and has multicomponents, multitargets, and multiapproaches. Amide alkaloids are important substance bases, and HJ is more suitable for treating inflammatory pain.

## 1. Introduction

Pain is a complex pathophysiological and psychological phenomenon that is commonly manifested in clinical practice, including pain sensation and the body's response to harmful stimulation. Conventionally, the 5 principles for managing pain proposed by the World Health Organization were followed. However, current medications for pain management have been limited due to inevitable adverse reactions. For example, the ceiling effect usually exists in the administration of nonsteroids or weak opioids that their analgesic effects would not be enhanced when their doses reach a certain degree, whereas higher doses lead to adverse reactions at high-risk level, instead of stronger therapeutic effects. Drug abuse or addiction is also an unavoidable public health issue [1–3]. Therefore, it is important to develop a candidate drug and alternative therapy for pain management.

As a typical Chinese herb applied in both medication and food, Huajiao (HJ) was first recorded in the Shen Nong's Classic of Materia Medica (*Shen Nong Ben Cao Jing*) in the Han dynasty, of which the amide alkaloids were the main bioactive components for analgesic effect. It was reported that the *α*-sanshool in HJ could decrease the frequency of body torsion, and fagaramide could remarkably relieve the pain induced by formalin and capsaicin [4]. Studies on the underlying mechanisms have revealed that fagaramide could develop the analgesic effect by suppressing the voltage-dependent Na^+^ channel in the A*δ* mechanic analgesic receptor and reducing the excitation output from the nociceptor [5]. Also, transient receptor potential (TRP)A1 and TRPV1, which belong to the TRP family, could be stimulated by hydroxy-alpha-sanshool in HJ to relieve pain [6]. HJ's anti-inflammatory activity has also been investigated, and its ethanol extracts were found to be able to suppress the stimulation from lipopolysaccharide to J774.1 macrophage and reduce the lipopolysaccharide/interferon-*γ*-induced infiltration of peritoneal macrophage via suppressing inducible nitric oxide synthase in mice [7]. Amide alkaloids in HJ are capable of reducing lipopolysaccharide-induced nitric oxide production in RAW264.7 macrophage [8]. HJ could be a potential candidate in pain management. Given that the material basis is complex with multicomponents, multitargets, and multiapproaches, the conventional pharmacological study might be insufficient to elucidate the mechanism and scientific connotation underlying the analgesic effects HJ.

As an essential part of systematic biology and bioinformatics, network pharmacology focuses on the intervention and regulation of diverse components of a single Chinese herb or a compound prescription for diseases. As it is integral, systematical, and interactive, the feature of network pharmacology is consistent with the therapeutic idea of holism and syndrome differentiation in traditional Chinese medicine. It has unique advantages in discovering the network of Chinese herbs with multicomponents, multitargets, and interactions at multilevels [9], exploring the synergetic behaviors of diverse components and the prescription compatibility connotation of Chinese herbs referred to the combination principles of traditional Chinese medicine [10, 11], predicting the active ingredients or component group under the potential biologic mechanisms [12], identifying the quality markers or toxicity quality markers to heighten the current unneutral quality control specification of traditional Chinese [13, 14], and investigating the variant biologic basis of a single herb with multiefficacy. Therefore, the present study aimed to clarify HJ's bioactive components in its analgesic effect and elucidate the underlying mechanism, which is further validated by semiflexible molecular docking. Our study would be helpful for the rationality and standardization of HJ application in clinical practice.

## 2. Methods

### 2.1. Database Establishment of Chemical Components of HJ

HJ's chemical constituents were obtained from the Traditional Chinese Medicine Systems Pharmacology Database and Analysis Platform (http://tcmspw.com/tcmsp.php) and published studies [15–18]. The structural formulas were formed using ChemBioDraw Ultra 14.0 (PerkinElmer, USA) and exported in the format as SYBYL2 (^*∗*^.mol 2).

### 2.2. Prediction and Screening of Potential Target

HJ's chemical components were uploaded to Swiss Target Prediction (http://www.swisstargetprediction.ch/) to predict potential targets and analyze the gastrointestinal absorption, skin permeation, and drug-likeness. The corresponding species were set as *Homo sapiens*. The probability score of predicted targets was resorted in a descending trend, and targets with a probability score (≥2 times the median) were selected as HJ's potential targets.

### 2.3. Disease-Target Database Establishment

The keyword “pain” was input into Online Mendelian Inheritance in Man (OMIM; http://www.omim.org/) to search genes related to pain. Pain-related genes and target proteins were selected, and the dataset was established after it was repeated, and false-positive genes were removed. Other human interactive target proteins were obtained from the Database of Interacting Proteins (DIP; http://dip.doe-mbi.ucla.edu/dip/Main.cgi).

### 2.4. Compound-Target-Disease Network Construction

The compound-target-disease network of HJ in relieving pain was constructed with interacting proteins and visualized using Cytoscape 3.6.0 (National Institute of General Medical Sciences, Bethesda, MD, USA), further analyzing the predicted potential target database with an established disease target database. The built-in module, NetworkAnalyzer, was utilized to analyze the characteristic topological parameters, including degree, betweenness centrality, and closeness centrality, for further screening the collected targets in HJ for pain relief, as previously reported [19] using following equations:

(1) $$\begin{array}{c} \text{degree}:K_{v}, \end{array}$$

where *v* was the number of links to node,

(2) $$\begin{array}{c} \text{betweenness centrality}:\Phi \left(v\right)=\sum_{s\neq v\neq t\in V}\frac{\sigma_{\text{st}}\left(v\right)}{\sigma_{\text{st}}}, \end{array}$$

where *σ_st_* was the number of the shortest paths between node *s* and *t* and *σ*st (*v*) was the number of the shortest paths passing through a node *v* out of *σ*st, and

(3) $$\begin{array}{c} \text{closeness centrality : }CC_{i}=\frac{N_{p}}{\sum_{j}L_{ij}}\;, \end{array}$$

where *N*_*p*_ was the total number of vertices in the graph and *L*_*ij*_ was the shortest path between vertices *i* and *j*.

Based on the rank of topological parameters in a descending trend, the top 20 compounds were screened. The related signaling pathways were enriched using the Database for Annotation, Visualization, and Integrated Discovery version 6.8 (DAVID; http://david.abcc.ncifcrf.gov/).

### 2.5. Docking between Compounds and Targets

The top 20 compounds in HJ were selected as candidates based on characteristic parameters in the network analysis. All candidates' information was transferred to the simplified molecular-input line-entry system, which was uploaded to Molinspiration Cheminformatics server (http://www.molinspiration.com/) to test their druggability (mainly including GPCR ligand, ion channel modulator, kinase inhibitor, nuclear receptor ligand, protease inhibitor, and enzyme inhibitor) using Predict Bioactivity button built-in Calculation of Molecular Properties and Prediction of Bioactivity module. The top 5 of the 20 compounds were selected as candidates for docking according to their druggability and bioactivity scores. The structures of small molecules were saved as mol.2 formats. MGLTools 1.5.6 was applied to add hydrogens, calculate electric charges, and merge nonpolar hydrogens of both small molecules and receptor p38*α* (PDB ID: 4KIQ, http://www.rcsb.org/structure/4KIQ) before being saved as pdbqt format. Active spots were confirmed according to the position of the originally inward ligand in p38*α* [20]. The Grid Box coordinates were set as 0.03, −4.767, and −2.807. In total, 8000 grids were confirmed (20 × 20 × 20), and the distance between each grid was 0.1 nm. The docking between small molecules with p38*α* was done using Autodock Vina (http://vina.scripps.edu/) [21]. Confirmation with the highest score was analyzed and visualized.

## 3. Results

### 3.1. Component-Target-Disease Network Establishment

**Figure 1:**
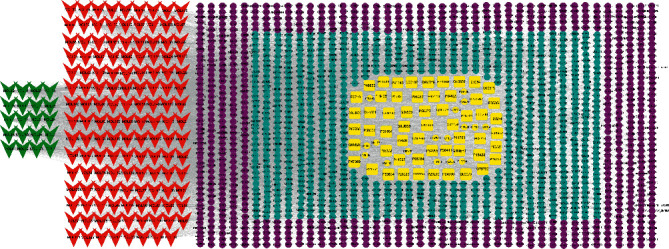
Component-target-disease network of pain relief using Huajiao (HJ). Red triangles indicate HJ components. Green triangles indicate top 20 components, which mainly include amide alkaloids. Peripheral purple circles indicate interactive targets. Internal fluorescent blue circles indicate predicted targets of HJ. Central yellow ovals indicate direct analgesic targets of HJ.

In total, HJ's 157 components were determined by literature retrieval and professional databases, including alkaloids, terpenoids, flavonoids, and fatty acids. A total of 746 predicted targets and 928 pain-related targets were obtained from OMIM and DIP. To facilitate the interpretation of delicate relationships between components and their potential targets in pain, an integrative component-target-disease network was used (Figure 1). The module Network Analyzer was used to calculate characteristic parameters, including degree, closeness centrality, and betweenness centrality, and the top 20 were screened. It was shown that HJ components with an analgesic effect were mainly amide alkaloids, which could be easily absorbed in the gastrointestinal tract with high bioavailability, indicating its potential property in drug likeness (Table 1). Considering HJ's frequent external use to destroy parasites and relieve pain and itching, skin permeation (log^*Kp*^) of 20 components was evaluated. Compared with the commonly externally used nonsteroidal, anti-inflammatory drug diclofenac (−4.96 cm/s) and scarcely nontransdermal cardiovascular drug ouabain (−10.94 cm/s), it was speculated that the skin permeation of 20 amide alkaloids was moderate. Collectively, HJ could relieve pain in conformity with both traditional oral administration and application by dermal permeation. Based on probability scores 2 times the median of the calculated parameters, 66 proteins with degree = 4, closeness centrality = 0.3, and betweenness centrality = 0.005 were identified as HJ's main targets for pathway enrichment analysis (Table 2).

### 3.2. Enrichment Analysis

**Figure 2:**
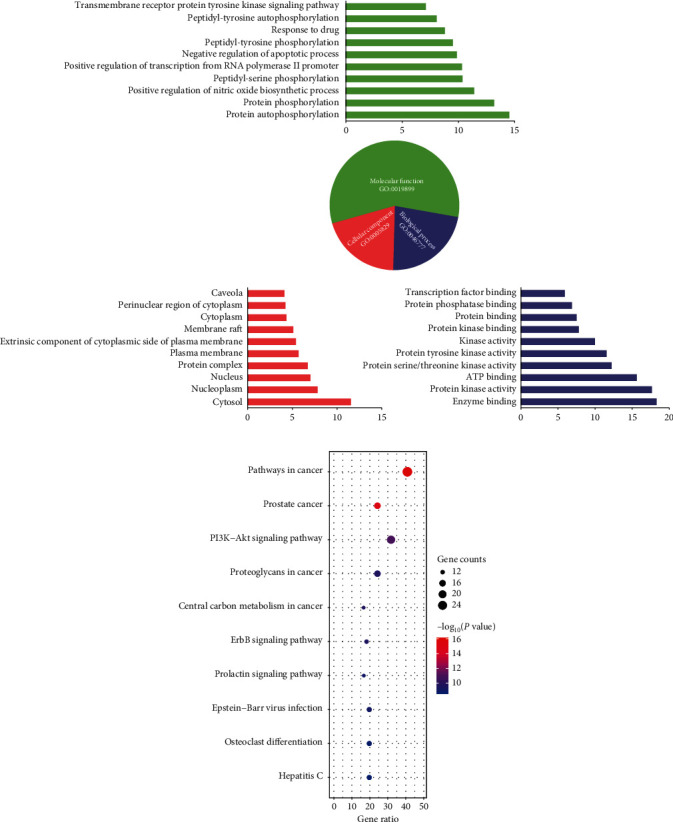
Enrichment analysis of Huajiao (HJ) in pain relief. (a) Gene ontology enrichment analysis of HJ targets. Biologic process (purple), molecular function (green), and cellular component (pink) accounted for 22.72%, 57.58%, and 31.82%, respectively. (b) Top 10 of 54 significantly enriched pathways of HJ with analgesic effect.

**Figure 3:**
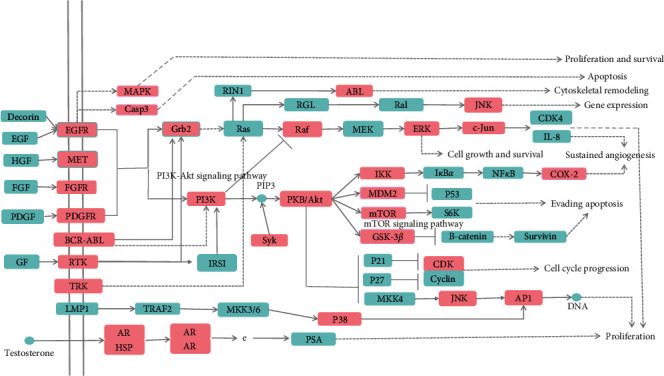
System map of Huajiao (HJ) in pain relief. Arrows indicate promotion and T-arrows indicate inhibition. Green squares represent targets in pathway, and pink squares represent HJ targets in relieving pain.

To explore the biologic procedure of relieving pain by HJ, the screened 66 targets were input into DAVID for the gene ontology (GO) enrichment analysis. In total, 257 enriching items were acquired (*P* < 0.05), including 307 biologic processes, 78 molecular functions, and 54 cell compositions. It was demonstrated that the main biologic processes involved in the analgesic effect of HJ were transcription factor binding, protein phosphatase binding, protein binding, protein kinase activity, kinase activity, protein tyrosine kinase activity, protein serine/threonine kinase activity, ATP binding, protein kinase activity, and enzyme binding. For molecular function, HJ exhibited an analgesic effect through the transmembrane receptor protein tyrosine kinase signaling pathway, protein autophosphorylation, protein phosphorylation, positive regulation of nitric oxide biosynthetic process, peptidyl-serine phosphorylation, positive regulation of transcription from RNA polymerase II promoter, negative regulation of the apoptotic process, peptidyl-tyrosine phosphorylation, response to the drug, and peptidyl-tyrosine autophosphorylation. The enrichment of cell composition indicated that HJ's targets were mainly distributed in the nucleoplasm, nucleus, protein complex, plasma membrane, an extrinsic component of the cytoplasmic side of the plasma membrane, membrane raft, cytoplasm, perinuclear region of cytoplasm, caveola, and cytosol (Figure 2(a)). Pathway enrichment based on the Kyoto Encyclopedia of Genes and Genomes (KEGG) showed that 41 pathways were involved in the analgesic effect of HJ, including pathways in cancer, the phosphoinositide 3-kinase- (PI3K-) protein kinase B (Akt) signaling pathway, prostate cancer, proteoglycans in cancer, mitogen-activated protein kinase (MAPK) signaling pathway, Ras signaling pathway, Epstein–Barr virus infection, osteoclast differentiation, and hepatitis C (Figure 2(b)). The pathways were integrated and visualized using KEGG Mapper. It was observed that there were 29 related targets, including MAPK, caspase 3, EGFR, MET, FGFR, PDGFR, BCR–ABL, RTK, TRK, Grb2, AR, HSP, PI3K, ABL, Jun-N-terminal kinase (JNK), Raf, extracellular signal-regulated kinase (ERK), c-Jun, Syk, PKB/Akt, IKK, MDM2, mTOR, GSK-3*β*, CDK, p38, AP1, and cyclooxygenase 2 (COX-2), which were mainly distributed in MAPK, PI3K-Akt-mTOR, and IKK-nuclear factor-*κ*B- (NF-*κ*B-) COX 2 and involved in the analgesic effect of HJ (Figure 3).

### 3.3. Molecular Docking of Bioactive Components of HJ

**Figure 4:**
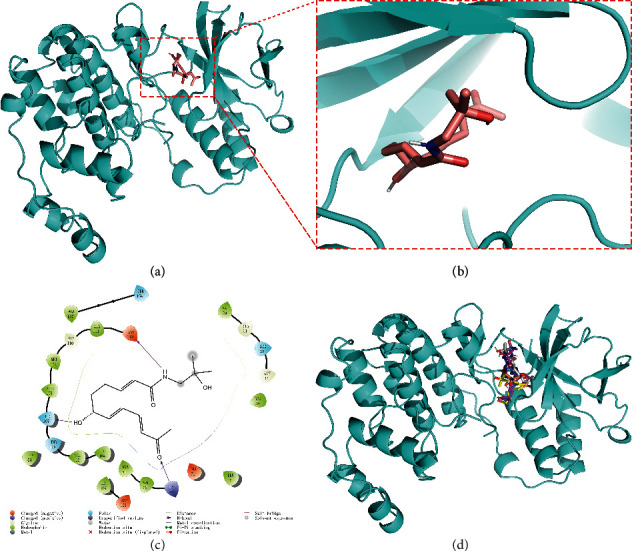
Molecular docking between the 5 amide alkaloids and p38*α*. (a) 3D mimic diagram of (6RS)-(2E,7E,9E)-6-hydroxy-N-(2-hydroxy-2-methylpropyl)-11-oxo-2,7,9-dodecatrienamide docking with p38*α*. (b) Partially enlarged view of 3D mimic diagram. (c) 2D mimic diagram of (6RS)-(2E,7E,9E)-6-hydroxy-N-(2-hydroxy-2-methylpropyl)-11-oxo-2,7,9-dodecatrienamide docking with p38*α*. (d) 3D mimic diagram of 5 amide alkaloids docking with p38*α*.

Based on the druggability test of the Swiss Institute of Bioinformatics, the following amide alkaloids were identified as the top 5 of 20 compounds (Table 3): (i) (6RS)-(2E,7E,9E)-6-hydroxy-N-(2-hydroxy-2-methylpropyl)-11-oxo-2,7,9-dodecatrienamide; (ii) (2E,7E,9E)-N-(2-hydroxy-2-methylpropyl)-11-ethoxy-6-hydroxy-dodeca-2,7,9-trienamide; (iii) (10RS,11RS)-(2E,6Z,8E)-10,11-dihydroxy-N-(2-hydroxy-2-methylpropyl)-2,6,8-dodecatrienamide; (iv) (2E,7E,9E)-N-(2-hydroxy-2-methylpropyl)-6-ethoxy-11-hydroxy-dodeca-2,7,9-trienamide; and (v) dioxo-2,7,9-dodecatrienamide. Importantly, p38, one of the major members in mitogen-activated protein kinase signaling pathway, is discovered to play a critical role in different pain states, as a key mediator modulating various signaling pathways in development and/or maintenance of pain [22–25]. Therefore, it was chosen as a candidate for molecular docking to further clarify the mechanism underlying pain intervention by HJ. Soft molecular docking between 5 amide alkaloids and p38*α* was accomplished using Autodock Vina. It was observed that the active site of p38*α* presented as a long narrow cavity that could fit all 5 amide alkaloids; the side with the amino of amide alkaloids was exposed outwards, and another side stretched into the hydrophobic cavity (Figure 4). It is generally considered that the absolute value of binding energy is higher, and the required free energy is lower in the mutual binding process. Simultaneously, it is a high probability and effectual binding if the absolute value of binding energy is greater than 6. Moreover, multiple hydrogen bonds were formed between 5 compounds and amino acids in the vicinity of the active site of p38*α*, and interactions facilitated the stable binding. This was also suggestive of high affinity between 5 compounds and p38*α* by hydrogen binding energy (Table 4) and could indicate that HJ relieves pain via binding with p38*α* to inhibit its activity.

## 4. Discussion

Pain, a complicated physical and mental activity, is a common symptom including pain sensation and pain reaction in clinic. Pathological pain includes inflammatory and neuropathological pain, which is a process of the manifestations of plastic change in neurons. There are concerns regarding the effectiveness and safety of pain management medications in clinical application. HJ, a common Chinese herbal medicine, has focused attention on its potential role in pain relief. Given its chemical complexity and diverse biofunction, the bioinformatics approach of network pharmacology integrated with soft molecular docking was performed to clarify HJ's bioactive components to exert an analgesic effect and explore the underlying mechanism. Our study suggested that amide alkaloids were the main components responsible for HJ's analgesic effect, with p38 considered the most important potential binding target.

Peripheral and central sensitivity are the pathological bases of pain generation, in which the intracellular signaling pathway activation plays a critical role in the underlying mechanism. MAPK, a serine/threonine-protein kinase in eukaryotic cells, is not only involved in inflammation and apoptosis but is also essential in mediating neuroplasticity and nerve function. It is a process that promotes the formation of hyperalgesia in both peripheral and central nerves, which contributes to pain sensation [26]. The MAPK cascade includes ERK, JNK/SAPK, p38 MAPK, and ERK5. These parallel signaling pathways are significantly associated with the formation, maintenance, and enhancement of pain. In the pathogenesis of pain, the peripheral nociceptive signal is imported into the spinal dorsal horn through the afferent nerve fiber, which causes the upregulation of excitatory amino acids and stress proteins. It leads to continuous depolarization of the nociceptive neuron with large amounts of calcium ion influx. The influx of calcium ion triggers intracellular calcium ion-dependent proteins, such as CaMK, PKA, and PKC. Generally, multiple and changed cellular signaling transduction pathways are involved in the MAPK signaling pathway. In particular, the p38 MAPK is a classic signaling pathway generating hyperalgesia in both peripheral and central nerves and induces various intracellular responses associated with neuropathic pain and other chronic pains. The p38 MAPK is strongly involved in inflammation and serves as an essential pathway producing proinflammatory factors in central glial cells, a critical target in intervening pain. Moreover, PI3K-Akt-mTOR is also involved in the occurrence and development of pain, either in synapse plasticity or in pain sensation facilitation after stimulation by pathological factors [27]. The activation of IKK-NF-*κ*B-COX-2 increases the production of inflammatory factors and COX-2, generating prostaglandins metabolized from arachidonic acid, which eventually contributes to pain [28].

## 5. Conclusions

HJ has an analgesic effect, and it could be used in pain management. The present study's findings indicate that HJ is more suitable for relieving inflammatory pain, such as bone fracture, muscle injury, and urgent intestinal contracture. Also, according to the theory in traditional Chinese medicine, the cold or heat property of HJ is different from distinct when used in pain management. That is, HJ could be applied to cure trauma with less concern about onset time. Given that our results were discovered *in silico* model, more studies based on *in vitro* or *in vivo* models should be encouraged to support our findings. Our study provides an integral view of HJ's bioactive chemical substances and its potential targets as the underlying mechanism for its analgesic effect, which is beneficial for both clinical medication in pain management and underlying mechanism elucidation.

## Figures and Tables

**Table 1:** Top 20 analgesic components of Huajiao.

No.	Name	Structure	Gastrointestinal absorption	log^*Kp*^ (cm/s)	Bioavailability score
1	(6RS)-(2E,7E,9E)-6-Hydroxy-N-(2-hydroxy-2-methylpropyl)-11-oxo-2,7,9-dodecatrienamide	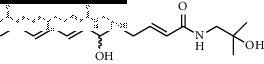	High	−7.73	0.55
2	(2E)-6,6-Dimethoxy-N-(2-hydroxy-2-methylpropyl)-2-hexenamide	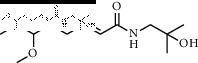	High	−7.50	0.55
3	(2E,7E,9E)-N-(2-Hydroxy-2-methylpropyl)-11-ethoxy-6-hydroxy-dodeca-2,7,9-trienamide	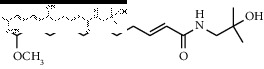	High	−7.41	0.55
4	(2E,4E,8Z,11E)-2'-Hydroxy-N-isobutyl-2,4,8,11-tetradecatetraenamide	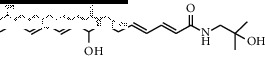	High	−7.41	0.55
5	Tetradecatetraenamide	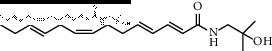	High	−5.56	0.55
6	N-[2-(3,4-Dimethoxyphenyl) ethyl]-3-phenyl-acrylamide	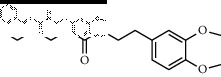	High	−6.23	0.55
7	Hydroxy-*α*-sanshool	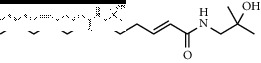	High	−5.90	0.55
8	Hydroxy-*γ*-sanshool	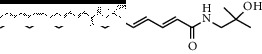	High	−5.61	0.55
9	(2E,7E,9E)-N-(2-Hydroxy-2-methylpropyl)-6-ethoxy-11-hydroxy-dodeca-2,7,9-trienamide	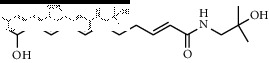	High	−7.41	0.55
10	Arnothianamide	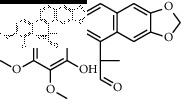	High	−5.66	0.55
11	(11RS)-(2E,7E,9E)-11-hyHydroxydroxy-N-(2-hydroxy-2-methylpropyl)-6-oxo-2,7,9-dodecatrienamide	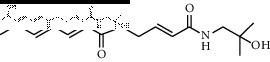	High	−7.78	0.55
12	(2E,6E,8E)-N-(2-Hydroxy-2-methylpropyl)-10-hydroxy-5-oxo-undeca-2,6,8-trienamide	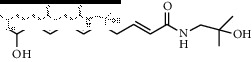	High	−7.90	0.55
13	(2E,4E,9E,11E)-N-(2-hydroxy-2-methypropyl)-8-hydroxy-13-oxo-2,4,9,11-tetradecatetraenamide	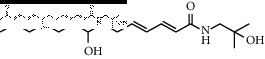	High	−7.44	0.55
14	(6RS,11RS)-(2E,7E,9E)-N-(2-Hydroxy-2-methylpropyl)-6,11-dioxo-2,7,9-dodecatrienamide	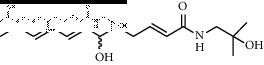	High	−7.71	0.55
15	*γ*-Sanshool	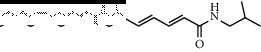	High	−4.52	0.55
16	Rutin	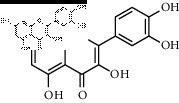	High	−7.05	0.55
17	Hydroxy-*β*-sanshool	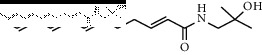	High	−5.90	0.55
18	(2E,4E,8Z,11Z)-N-(2-Hydroxy-2-methylpropyl)-2,4,8,11-tetradecatetraenamide	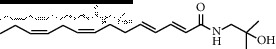	High	−5.56	0.55
19	3,5,6-Trihydroxy-7,4´-dimethoxy flavone	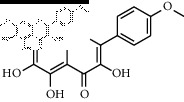	High	−6.31	0.55
20	*β*-Sanshool	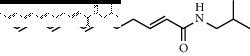	High	−4.82	0.55

**Table 2:** The dataset of 66 proteins for pathway enrichment analysis.

ID	Protein name	Degree	Closeness centrality	Betweenness centrality
P42574	Caspase-3	23	0.31	0.01
Q13547	Histone deacetylase 1	20	0.31	0.01
P78527	DNA-dependent protein kinase catalytic subunit	21	0.32	0.01
P42338	Phosphatidylinositol 4,5-bisphosphate 3-kinase catalytic subunit beta isoform	13	0.31	0.01
P05412	Transcription factor AP-1	16	0.33	0.01
P23219	Prostaglandin G/H synthase 1	44	0.34	0.01
Q16665	Hypoxia-inducible factor 1-alpha	22	0.30	0.01
P30305	M-phase inducer phosphatase 2	24	0.33	0.01
P49336	Cyclin-dependent kinase 8	26	0.32	0.01
P07900	Heat shock protein HSP 90-alpha	41	0.37	0.02
P50613	Cyclin-dependent kinase 7	13	0.32	0.01
P04629	High affinity nerve growth factor receptor	20	0.33	0.01
P23458	Tyrosine-protein kinase JAK1	28	0.34	0.01
P62993	Growth factor receptor-bound protein 2	20	0.32	0.01
O60674	Tyrosine-protein kinase JAK2	30	0.34	0.01
Q99558	Mitogen-activated protein kinase kinase kinase 14	26	0.33	0.01
P42345	Serine/threonine-protein kinase mTOR	25	0.33	0.01
P35354	Prostaglandin G/H synthase 2	51	0.34	0.01
P09874	Poly [ADP-ribose] polymerase 1	40	0.35	0.02
P06213	Insulin receptor	26	0.33	0.01
P31749	RAC-alpha serine/threonine-protein kinase	22	0.33	0.01
P12931	Proto-oncogene tyrosine-protein kinase Src	28	0.36	0.01
P49841	Glycogen synthase kinase-3 beta	31	0.35	0.01
Q96GD4	Aurora kinase B	19	0.32	0.01
P00918	Carbonic anhydrase 2	76	0.37	0.01
P18031	Tyrosine-protein phosphatase nonreceptor type 1	53	0.35	0.01
P22303	Acetylcholinesterase	65	0.36	0.01
P11511	Aromatase	83	0.36	0.02
P11362	Fibroblast growth factor receptor 1	18	0.30	0.01
P09619	Platelet-derived growth factor receptor beta	23	0.33	0.01
P04049	RAF protooncogene serine/threonine-protein kinase	35	0.33	0.02
O15111	Inhibitor of nuclear factor kappa-B kinase subunit alpha	23	0.32	0.01
O14920	Inhibitor of nuclear factor kappa-B kinase subunit beta	27	0.34	0.01
O14965	Aurora kinase A	29	0.34	0.01
P24941	Cyclin-dependent kinase 2	41	0.34	0.01
Q07869	Peroxisome proliferator-activated receptor alpha	58	0.34	0.01
P06493	Cyclin-dependent kinase 1	35	0.34	0.01
P09917	Arachidonate 5-lipoxygenase	55	0.36	0.01
P04150	Glucocorticoid receptor	54	0.36	0.01
P43405	Tyrosine-protein kinase SYK	25	0.34	0.01
P56817	Beta-secretase 1	36	0.36	0.01
P0DMS8	Adenosine receptor A3	35	0.35	0.01
P30542	Adenosine receptor A1	47	0.36	0.01
Q16539	Mitogen-activated protein kinase 14	38	0.37	0.01
P45983	Mitogen-activated protein kinase 8	25	0.33	0.01
Q00987	E3 ubiquitin-protein ligase Mdm2	26	0.34	0.01
P05067	Amyloid-beta precursor protein	29	0.34	0.02
P00533	Epidermal growth factor receptor	57	0.37	0.03
P32246	C–C chemokine receptor type 1	19	0.31	0.01
P28482	Mitogen-activated protein kinase 1	29	0.34	0.01
P08581	Hepatocyte growth factor receptor	29	0.34	0.01
P06401	Progesterone receptor	53	0.35	0.01
P03372	Estrogen receptor	66	0.34	0.02
O43570	Carbonic anhydrase 12	40	0.35	0.01
P00915	Carbonic anhydrase 1	62	0.37	0.01
P35968	Vascular endothelial growth factor receptor 2	45	0.36	0.02
P00519	Tyrosine-protein kinase ABL1	24	0.33	0.01
P28845	Corticosteroid 11-beta-dehydrogenase isozyme 1	67	0.37	0.01
O00519	Fatty-acid amide hydrolase 1	47	0.35	0.01
P41594	Metabotropic glutamate receptor 5	34	0.36	0.01
P10275	Androgen receptor	108	0.38	0.05
P37231	Peroxisome proliferator-activated receptor gamma	39	0.34	0.01
O75762	Transient receptor potential cation channel subfamily A member 1	38	0.35	0.01
Q8NER1	Transient receptor potential cation channel subfamily V member 1	49	0.36	0.01
P34972	Cannabinoid receptor 2	57	0.35	0.01
P21554	Cannabinoid receptor 1	40	0.36	0.01

**Table 3:** Druggability information of 5 amide alkaloids.

No.	GPCR ligand	Ion channel modulator	Kinase inhibitor	Nuclear receptor ligand	Protease inhibitor	Enzyme inhibitor	OWAR (log *P*)	TPSA	Lipinski rules
1	0.31	0.38	−0.23	0.41	0.3	0.53	0.83	86.62	√
2	0.41	0.4	0	0.42	0.31	0.43	1.64	78.79	√
3	0.44	0.38	0	0.4	0.29	0.45	1.54	89.78	√
4	0.41	0.36	−0.01	0.33	0.23	0.46	1.64	78.79	√
5	0.43	0.41	−0.05	0.39	0.24	0.5	1.02	89.78	√

GPCR, G-protein coupled receptors; OWAR, octyl alcohol/water allocation ratio; TPSA, topological polar surface area.

**Table 4:** Binding energy and hydrogen bond sites of 5 amide alkaloid with p38*α*.

No.	Binding energy (kcal/mol)	Hydrogen bond of amino acid
1	−7.3	LYS 53, HID 107, ASP 112
2	−6.5	ASP 112, THR 106, ASP 168
3	−7.9	PHE 169, GLY 110, ASP 112
4	−7.6	GLU 71, THR 106, ASP 112
5	−7.6	THR 106, SER 154, PHE 169

## Data Availability

The datasets used and analyzed during the current study are available from the corresponding author on reasonable request.
